# Prefrontal Structural Asymmetry Mediates Body Mass Index and Treatment Response in Major Depressive Disorder

**DOI:** 10.1155/da/9924894

**Published:** 2026-05-25

**Authors:** Kaini Qiao, Zhenxiang Zang, Yun Wang, Xiongying Chen, Ke Liu, Zhifang Zhang, Rui Liu, Dong Li, Yanting Luo, Le Xiao, Xuequan Zhu, Jingjing Zhou, Zhi Yang, Gang Wang

**Affiliations:** ^1^ National Clinical Research Center for Mental Disorders & National Center for Mental Disorders, Beijing Anding Hospital, Capital Medical University, Beijing, China, ccmu.edu.cn; ^2^ Beijing Key Laboratory of Intelligent Drug Research and Development for Mental Disorders, National Clinical Research Center for Mental Disorders, National Center for Mental Disorders, Beijing Anding Hospital, Capital Medical University, Beijing, China, ccmu.edu.cn

**Keywords:** BMI, brain asymmetry, major depressive disorder, prefrontal cortex

## Abstract

**Background:**

Elevated body mass index (BMI) predicts poor treatment response in major depressive disorder (MDD), yet the neurobiological mechanisms underlying this association remain poorly understood. Cortical thickness asymmetry has been proposed as a structural correlate of affective regulation and may represent a pathway through which metabolic factors influence treatment outcomes. We investigated whether BMI‐related alterations in prefrontal cortical asymmetry mediate antidepressant treatment resistance in MDD.

**Methods:**

We analyzed baseline structural MRI (sMRI) and 12‐week clinical outcome data from 312 adults with MDD across a discovery cohort (*n* = 107) and an independent replication cohort (*n* = 205). Treatment response was operationalized as absolute reduction in Hamilton depression rating scale (HAMD‐17) scores. Cortical thickness asymmetry indices were derived from regional parcellations. Associations between BMI and asymmetry were examined using linear regression models; mediation analyses tested whether BMI‐related asymmetry statistically mediates the link between higher BMI and reduced treatment response. Sex‐stratified analyses were conducted to identify divergent pathways.

**Results:**

Across both cohorts, higher BMI was consistently associated with greater leftward cortical thickness asymmetry in the prefrontal cortex (PFC). This structural asymmetry significantly mediated the relationship between BMI and poorer treatment response. Sex‐stratified analyses revealed additional female‐specific mediation through prefrontal opercular regions, with no corresponding effect in males. Transcriptomic annotation of implicated regions identified enrichment for genes involved in metabolic and cytoplasmic signaling pathways.

**Conclusions:**

BMI‐associated leftward prefrontal asymmetry statistically mediates antidepressant resistance in MDD via both a sex‐shared structural pathway and a female‐specific opercular circuit. These findings suggest that metabolic factors may influence treatment outcomes partly through hemispheric structural imbalance in prefrontal regions and position cortical thickness asymmetry as a candidate neuroimaging biomarker for patient stratification in precision psychiatry.

## 1. Introduction

Major depressive disorder (MDD) ranks among the leading causes of disability worldwide, yet its neurobiological basis remains incompletely understood. While subtle brain structural alterations are believed to play a role [1], neuroimaging findings are often inconsistent—likely due to clinical heterogeneity and comorbid conditions such as elevated body mass index (BMI). High BMI and MDD share a complex, bidirectional relationship [2, 3], driven by overlapping mechanisms including genetic predispositions, immune–inflammatory responses, neuroendocrine and metabolic imbalances, gut microbiome disruptions, and behavioral factors [4–6]. Clinically, this comorbidity is critical: individuals with higher BMI often exhibit resistance to antidepressant treatment [7], a finding corroborated by our previous work showing attenuated symptom reduction in patients with higher BMI [8].

Neural alterations may serve as the biological bridge between depression and high BMI. Both conditions are associated with brain structural alterations in regions governing cognitive control and reward processing [9, 10], particularly the prefrontal cortex (PFC) [11]. We hypothesize that BMI‐related morphological changes in the PFC may compromise treatment efficacy in MDD. Given that prefrontal cortical function relies on coordinated hemispheric specialization, we specifically focus on altered brain asymmetry as a marker of inter‐hemispheric structural balance. Lateralization is fundamental to emotional functioning, and its disruption is implicated in various psychiatric conditions [12–14].

Converging evidence indicates that cortical thickness asymmetry may reflect the neurobiological intersection of high BMI and MDD. A higher BMI has been linked to increased thickness in the left frontal lobe relative to the right, supporting the “right brain hypothesis” of obesity, which posits that right hemispheric deficits contribute to dysregulated eating behaviors [15, 16]. Similarly, aberrant frontal asymmetry is a recurrent finding in MDD [17–19]. This is particularly relevant given that non‐invasive brain stimulation (NIBS) protocols for depression often aim to restore hemispheric balance—for example, by enhancing left or inhibiting right dorsal PFC activity [20, 21]. Understanding how BMI influences this asymmetry could therefore inform personalized treatment strategies, particularly for patients with comorbid obesity and depression.

In this study, we examined whether cortical thickness asymmetry mediates the negative effect of BMI on antidepressant treatment response. Using a discovery‐replication design, we (1) characterized the relationship between BMI and cortical thickness asymmetry, (2) assessed the impact of BMI on treatment outcomes (HAMD‐17 change at week 12), and (3) conducted mediation analyses to test whether frontal asymmetry patterns explain the link between high BMI and treatment resistance. Finally, we explored shared genetic correlates to identify potential molecular pathways linking structural asymmetry to treatment outcomes in MDD.

## 2. Materials and Methods

### 2.1. Participants

This study included two cohorts. Cohort 1 served as a discovery dataset, comprising 107 patients with MDD enrolled in the “Appropriate Technology Study of MDD Diagnosis and Treatment Based on Objective Indicators and Measurement [22].” Cohort 2 served as a replication dataset, comprising 205 MDD patients from the PROUD study (Prospective Cohort Study of Depression) [23]. All analyses were conducted separately for each cohort, and results from both cohorts are presented in the main text. To minimize developmental effects on cortical thickness, only participants aged 22 years or older were included in this study [24].

All patients were recruited and screened at the Beijing Anding Hospital, Capital Medical University. The two cohorts shared largely consistent inclusion and exclusion criteria, with minor study‐specific differences. Common inclusion criteria were the following: Age between 18 and 65 years, a diagnosis of MDD–based on the DSM‐IV or DSM‐5 criteria, a score of ≥14 on the Chinese version of the 17‐item Hamilton depression rating scale (HAMD‐17) at screening or baseline, at least primary school education with the ability to understand the assessment scales, and no antidepressant use in the 14 days prior to enrollment, or use of antidepressants on fewer than 7 days within that 14‐day period. The common exclusion criteria included a current or past diagnosis of other psychotic disorders or substance abuse, severe or uncontrolled systemic diseases, MRI contraindications, and pregnancy or lactation. Cohort‐specific inclusion and exclusion criteria are detailed in Supporting Information 1: Table S1.

This study protocol was approved by the Ethics Committee of Beijing Anding Hospital, Capital Medical University (Approval nos. 2017‐24 and 2022‐14). Written informed consent was obtained from all participants.

### 2.2. Demographic Information and Assessment of BMI

Baseline assessment included demographic data, a physical examination, and MRI scans. Demographic variables comprised age and sex. Height and weight were measured during the physical examination, and BMI was calculated as body weight in kilograms divided by height in meters squared (kg/m^2^). BMI categories were defined according to the recommendations of the Working Group on Obesity in China [25]: underweight (BMI <18.5), normal weight (18.5 ≤ BMI <24.0), overweight (24.0 ≤ BMI <28.0), and obesity (BMI ≥28.0). Summary statistics for demographic characteristics and BMI are shown in Table 1.

**Table 1:** Demographic characteristics and clinical variables.

Variable	Discovery dataset (*n* = 107)	Replication dataset (*n* = 205)
Demographic variables		
Age (mean ± SD)	28.46 ± 5.37	29.48 ± 6.95
Gender (M/F)	36/71	62/143
BMI (mean ± SD)	21.77 ± 3.60	22.68 ± 3.53

Clinical variables		
HAMD‐17 score baseline (mean ± SD)	21.25 ± 4.37 (*n* = 101)	20.31 ± 3.90 (*n* = 204)
HAMD‐17 score week 12 (mean ± SD)	9.25 ± 5.93 (*n* = 68)	8.28 ± 5.61 (*n* = 178)
HAMD‐17 change (mean ± SD)	11.83 ± 6.40 (*n* = 65)	12.08 ± 6.05 (*n* = 177)

Abbreviation: HAMD‐17, Hamilton rating scale for depression (17‐item).

### 2.3. Lifestyle and Socioeconomic Variables

To account for potential confounds, we included a comprehensive set of lifestyle and socioeconomic covariates known to influence both metabolic health and antidepressant treatment response. Lifestyle variables were measured as follows: (1) dietary patterns, assessed via a food frequency questionnaire quantifying the intake (times per week) of high‐fat, high‐sugar, high‐salt, and probiotic‐rich foods; (2) physical activity, measured by self‐reported weekly duration and stratified into ordinal categories (≤10, 11–20, 21−30, 31–60, and >60 min per week); and (3) sleep duration, dichotomized into adequate (≥7 h) vs. inadequate (<7 h) sleep based on established clinical cutoffs. Socioeconomic status was proxied by educational attainment, coded as an ordinal variable. These variables were selected to control for variance attributable to environmental and behavioral factors.

### 2.4. Depressive Symptoms and Treatment Response

Symptom severity was assessed at baseline and at week 12 using the HAMD‐17. Treatment response was defined as the absolute reduction in HAMD‐17 score from baseline to week 12, with larger reductions indicating greater clinical improvement. Descriptive statistics for symptom severity and treatment response are presented in Table 1. In addition, the duration of the current depressive episode was recorded as a continuous variable.

### 2.5. Structural MRI Acquisition, Preprocessing, and Quality Control

The structural MRI (sMRI) data were acquired using a 3.0T Siemens Prisma MRI scanner with a 64‐channel head coil. T1‐weighted images were obtained using the magnetization‐prepared rapid acquisition gradient echo (MPRAGE) sequence, and earplugs were provided to attenuate scanner noise exposure. Acquisition parameters were consistent across participants, with minor variations in echo time, flip angle, and slice orientation (Supporting Information 2: Table S2). These variations are within typical ranges and were modeled as random effects in statistical analyses. An experienced neuroradiologist reviewed all MRI scans to exclude major artifacts and other abnormalities.

The data from both cohorts were preprocessed using the PhiPipe procedure [26]. T1‐weighted structural images were processed with FreeSurfer (version 7.3.2) and advanced normalization tools (ANTs, version 2.4.4). Preprocessing steps included skull stripping, tissue segmentation, surface reconstruction, and anatomical parcellation using FreeSurfer’s recon‐all. These steps were followed by nonlinear registration of the T1 images to the MNI152 T1 brain template using ANTs. Finally, quality control images for brain extraction, tissue segmentation, parcellation, and registration were visually inspected for accuracy.

### 2.6. Asymmetry Index Calculation

For each bilaterally paired brain region, the asymmetry index (AI) was calculated using the left (*L*) and right (*R*) hemispheric measurements with the following formula:

AI=L−RL+R.

This formula ensures that the index is appropriately scaled based on the magnitude of the bilateral measures. The AI was calculated for cortical thickness.

### 2.7. Examining the Association Between BMI and Brain Asymmetry

We utilized BMI as a continuous variable to enhance sensitivity and align with established practices in previous studies [27], rather than using categorical classifications. Linear mixed modeling was performed using the nlme package (version 3.1‐164) in R (version 4.3.3), with lateralized indices derived from cortical thickness as the dependent variables. The initial full model for each brain measure included BMI, age, sex, total intracranial volume (ICV), and a BMI‐by‐sex interaction term as fixed effects. To account for variability related to scanner parameters, models also incorporated a random effect for acquisition protocol. If the BMI‐by‐sex interaction was statistically significant, the model was interpreted by examining sex‐stratified simple slopes. For models without a significant interaction, the main effect of BMI from the reduced model was reported.

Effect sizes for BMI–brain measure associations were calculated as partial correlation coefficients (partial *r*), along with 95% confidence intervals (CIs), derived from the model coefficients and their standard errors (SE). To account for multiple comparisons, all *p*‐values were adjusted using the false discovery rate (FDR) method, with adjusted *p*‐values reported at a significance level of *α* = 0.05. The normality of model residuals was assessed using QQ plots, and multicollinearity was examined using the variance inflation factor (VIF) for all predictor variables, with a maximum value of 2.233, indicating no concerning multicollinearity. Complete computer code for these analyses is available upon request.

### 2.8. BMI Associations With Treatment Response

We examined the associations between baseline BMI and treatment response (HAMD‐17 total score reduction from baseline to week 12). Associations were evaluated using linear models, both unadjusted and adjusted for selective serotonin reuptake inhibitor (SSRI) dosage per kilogram of body weight and baseline HAMD‐17 scores. All analyses were controlled for age and sex. In both models, we included a BMI‐by‐sex interaction term to test for potential sex differences in the BMI–treatment response relationship. If the interaction term was statistically significant (*p* < 0.05), we conducted simple slope analyses separately for males and females. If the interaction was not significant, the term was removed, and the main effect of BMI was interpreted.

### 2.9. Associations Between BMI‐Related Brain Asymmetry and Treatment Response

We examined whether BMI‐related cortical asymmetry was associated with treatment response. To minimize multiple comparisons and ensure logical consistency, we restricted this analysis to the specific brain regions that demonstrated a significant association with BMI in the preceding step, including both regions with a significant main effect of BMI and regions with a significant BMI‐by‐sex interaction. Consequently, in the discovery dataset, we evaluated the association between treatment response and the asymmetry of the regions identified as BMI‐associated. Similarly, in the replication dataset, we evaluated the regions identified in that cohort. For each region, we fitted a linear mixed model with treatment response as the outcome. The model included cortical asymmetry as the predictor, with age, sex, and an asymmetry‐by‐sex interaction term as fixed effects. Random intercepts were included for the protocol to account for acquisition variability. Associations were evaluated with and without adjustment for baseline HAMD‑17 scores; adjusted results are reported in the tables.

### 2.10. Mediation Analysis

Mediation analyses tested whether cortical thickness asymmetry mediates the relationship between BMI and treatment response (change in HAMD‐17 scores), building on a substantial body of literature—including our prior study using a larger sample (*n* = 202) from the same discovery cohort [8]. The current findings further corroborate this relationship.

All mediation analyses used the causal mediation framework implemented in the mediation package (version 4.5.0) in R (version 4.3.3). We employed a dual analytical approach. First, in line with traditional criteria [28], we examined the component relationships necessary for mediation: (1) BMI was a significant predictor of cortical thickness asymmetry (the mediator); (2) the mediator significantly predicted treatment response; and (3) an attenuation of BMI’s direct effect on the outcome when the mediator is included. Second, following modern methodological recommendations [29–34], our primary inferential test focused on the indirect effect itself (a × b path).

To ensure robustness, we utilized 5000 bootstrap samples generated by random selection with replacement. Statistical significance of the indirect effect was determined by 95% CIs that did not include zero. All analyses were adjusted for age and sex. For visualization purposes, we used structural equation modeling (SEM) with the lavaan package (0.6.19) to estimate standardized path coefficients and create path diagrams.

### 2.11. Gene Association and Enrichment Analysis of Significant Mediating Regions

To explore molecular underpinnings, we leveraged Allen Human Brain Atlas (AHBA) data mapped to Desikan‐Killiany (DK) atlas regions using the abagen toolbox [35]. Focusing on regions that emerged as significant mediators, we calculated lateralization indices for gene expression and used one‐sample *t*‐tests to identify genes with significant hemispheric asymmetry. After Bonferroni correction, we identified asymmetrically expressed genes, with 432 genes overlapping between regions.

We next examined overlap with MDD‐associated transcriptomic signatures reported by Gandal et al. [36] and found 48 overlapping genes. Gene enrichment analysis was performed using DAVID (version 6.8), with significance defined as Bonferroni‐corrected *p*  < 0.05.

### 2.12. Control of Confounding Variables

To effectively control for potential confounders, we employed specific strategies to handle variable types, particularly addressing the multicollinearity among dietary measures. Dietary variables (high‐fat, high‐sugar, high‐salt, and probiotic consumption frequencies) were highly inter‐correlated. Therefore, we used principal component analysis (PCA) for data reduction, transforming these variables into orthogonal components that capture underlying dietary patterns without multicollinearity. The first two components (PC1 and PC2) were retained, which together explained 76.8% of the total variance. PC1 (eigenvalue = 2.037, 50.9% of variance) represented a general high‐frequency diet pattern, with positive loadings from high‐fat (0.573), high‐sugar (0.524), and high‐salt (0.613) foods. PC2 (eigenvalue = 1.037, 25.9% of variance) primarily represented probiotic consumption, indicated by a strong negative loading (−0.922) (Supporting Information 3: Figure S1). These components, along with physical activity, sleep duration, educational level, and illness duration (entered as described previously), were included in a comprehensive adjusted model in the replication dataset to rigorously control for confounding.

## 3. Results

### 3.1. BMI Shows Both Sex‐Specific and Sex‐Common Associations With Brain Asymmetry

**Figure 1:**
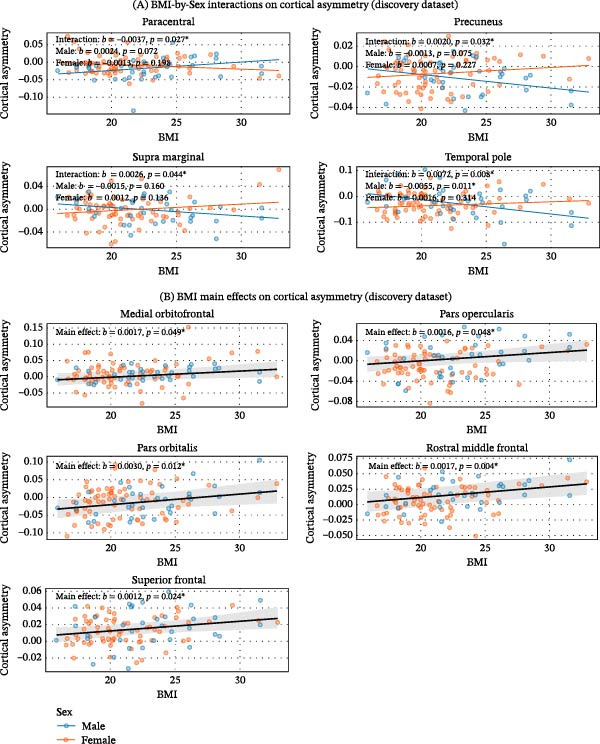
Associations between BMI and cortical thickness asymmetry in the discovery dataset. (A) BMI‐by‐sex interactions. Each subplot shows sex‐specific regression lines (blue = male, orange = female) and raw data (semitransparent). Annotations display interaction *b* and *p*‐value, and simple slopes per sex. (B) BMI main effects. Each subplot shows the single regression line (gray) with 95% confidence band and raw data colored by sex (visual aid only). Annotations report *b* and *p*‐value.  ^∗^ *p* < 0.05.

**Figure 2:**
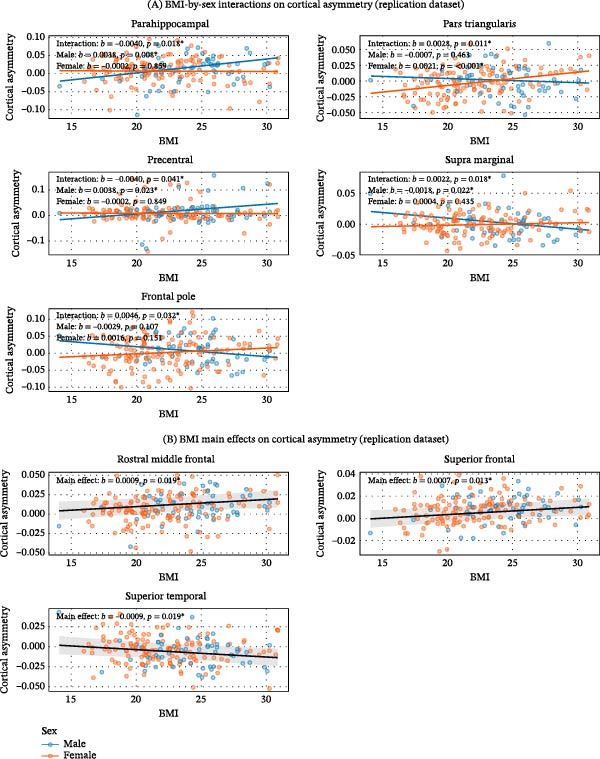
Associations between BMI and cortical thickness asymmetry in the replication dataset. (A) BMI‐by‐sex interactions. Sex‑specific regression lines (blue = male, orange = female) and raw data (semitransparent). Annotations show interaction *b* and *p*‑value, and simple slopes per sex. (B) BMI main effects. Single regression line (gray) with 95% confidence band and raw data colored by sex (visual aid only). Annotations report *b* and *p*‑value.  ^∗^ *p* < 0.05.

Interaction testing revealed significant BMI‐by‐sex interactions in multiple regions across both cohorts. In the discovery dataset, significant interactions were found for the paracentral, precuneus, supramarginal, and temporal pole (Figure 1A). Simple slope analyses showed a significant negative association in males for the temporal pole. In the replication dataset, significant interactions were confirmed for the parahippocampal, pars triangularis, precentral, supramarginal, and frontal pole (Figure 2A). Follow‐up simple slope analysis revealed distinct, sex‐specific associations. Sex‐specific simple slopes are fully reported in Supporting Information 4: Table S3 and Supporting Information 5: Table S8.

For all other brain regions examined where no significant BMI‐by‐sex interaction was observed, we interpreted the main effect of BMI, representing its sex‐averaged association with asymmetry. In the discovery dataset, higher BMI was associated with greater cortical thickness asymmetry (leftward shift) in the medial orbitofrontal, pars opercularis, pars orbitalis, rostral middle frontal, and superior frontal regions (Figure 1B). In the replication dataset, a similar pattern across sexes emerged: BMI showed positive associations with asymmetry in the rostral middle frontal and superior frontal regions (Figure 2B). In addition, BMI showed positive associations with asymmetry in the superior temporal region. Full statistical results are provided in Supporting Information 6: Table S4 and Supporting Information 7: Table S9.

### 3.2. Negative Association Between BMI and Treatment Response

**Figure 3:**
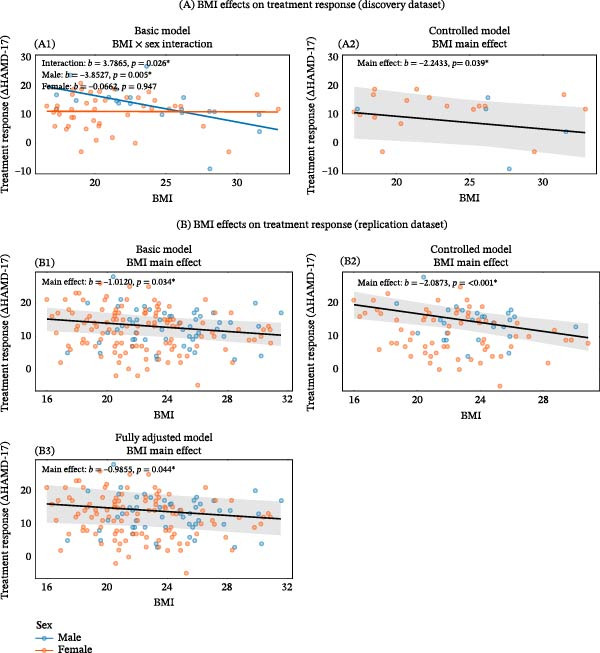
Associations between BMI and treatment response in the discovery and replication datasets. (A) Discovery dataset. (A1) Unadjusted: significant BMI‑by‑sex interaction. (A2) Fully adjusted (baseline HAMD‑17, dose/kg): interaction non‑significant; significant main effect of BMI is plotted. (B) Replication dataset. (B1) Unadjusted (age, sex): significant BMI main effect. (B2) Adjusted for baseline HAMD‑17 and dose/kg: significant BMI main effect. (B3) Additionally adjusted for lifestyle covariates: significant BMI main effect.  ^∗^ *p* < 0.05.

In the discovery dataset, a significant BMI‐by‐sex interaction was observed in the unadjusted model (*b* = 3.787, *p* = 0.0258); simple slopes revealed a strong negative effect in males but not in females. This interaction was not retained after covariate adjustment. In the replication dataset, no interaction was significant in any model, and a consistent negative main effect of BMI on treatment response was observed across all adjustment levels. Full details are provided in Supporting Information 8: Table S5 and Supporting Information 9: Table S10, and Figure 3.

### 3.3. Associations Between BMI‐Related Frontal Asymmetry and Treatment Response

**Figure 4:**
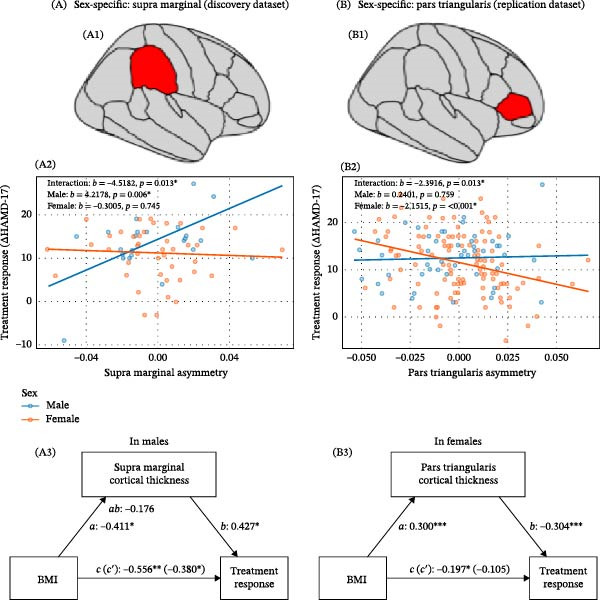
Sex‑specific mediation effects of cortical asymmetry on BMI–treatment response relationship. (A) Discovery dataset: (A1) Supramarginal gyrus. (A2) Significant interaction between cortical asymmetry and sex on treatment response. (A3) Mediation analysis in males: indirect effect nonsignificant; direct effect of BMI significant. (B) Replication dataset: (B1) Pars triangularis. (B2) Significant interaction between cortical asymmetry and sex on treatment response. (B3) Mediation analysis in females: significant indirect effect of BMI on treatment response via pars triangularis asymmetry. Path definitions: *c* = total effect of BMI; *a* = effect of BMI on asymmetry; *b* = effect of asymmetry on treatment response; *c*′ = direct effect of BMI; *ab* = indirect effect. For Subfigure (A), ab is non‐significant and *c*′ is significant; for Subfigure (B), *ab* is significant.  ^∗^ *p* < 0.05;  ^∗∗^ *p* < 0.01;  ^∗∗∗^ *p* < 0.001.

Significant asymmetry‐by‐sex interactions were identified for the supramarginal gyrus (discovery) and pars triangularis (replication). Simple slope analyses revealed sex‐specific effects: in discovery males, greater supramarginal gyrus asymmetry predicted greater response; in replication females, greater pars triangularis asymmetry predicted poorer response. These effects remained significant after adjusting for baseline symptom severity. Full details are provided in Supporting Information 10: Table S6 and Supporting Information 11: Table S11, and Figure 4.

**Figure 5:**
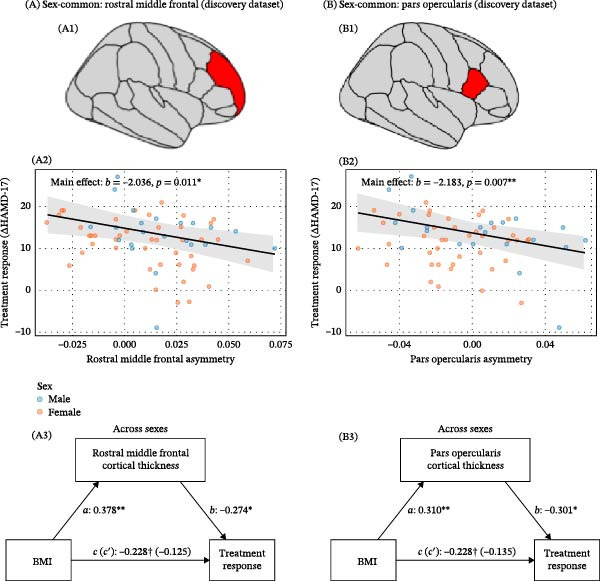
Sex‐common mediation effects of cortical asymmetry on the BMI–treatment response relationship. (A) Discovery dataset: (A1) Rostral middle frontal gyrus. (A2) Significant main effect of cortical asymmetry on treatment response. (A3) Mediation analysis across sexes: significant indirect effect of BMI on treatment response via rostral middle frontal asymmetry. (B) Discovery dataset: (B1) Pars opercularis. (B2) Significant main effect of cortical asymmetry on treatment response. (B3) Mediation analysis across sexes: significant indirect effect of BMI on treatment response via pars opercularis asymmetry. Path definitions: *c* = total effect of BMI; *a* = effect of BMI on asymmetry; *b* = effect of asymmetry on treatment response; *c*′ = direct effect of BMI. For Subfigures (A, B), *ab* is significant.  ^∗^ *p* < 0.05;  ^∗∗^ *p* < 0.01.

For regions without significant sex interactions, main effects of asymmetry were examined. Greater asymmetry in the rostral middle frontal region was associated with poorer treatment response (*b* = −2.036, SE = 0.78, *t*(60) = −2.62, *p* = 0.0112). Similarly, asymmetry in the pars opercularis showed a negative association with treatment response (*b* = −2.183, SE = 0.78, *t*(60) = −2.81, *p* = 0.0068). Both associations remained significant after controlling for baseline symptom severity. Full details are provided in Supporting Information 10: Table S6 and Supporting Information 11: Table S11, and Figure 5.

### 3.4. Mediating Effects of BMI‐Related Frontal Asymmetry

Mediation analyses were conducted to explore potential mechanistic pathways. In the discovery dataset, two prefrontal regions exhibited significant indirect effects across sexes. Cortical asymmetry in the rostral middle frontal region significantly mediated the relationship between BMI and treatment response (ACME: *b* = −0.103, 95% CI [−0.217, −0.01], *p* = 0.022). Similarly, asymmetry in the pars opercularis also approached significance as a mediator (ACME: *b* = −0.093, 95% CI [−0.232, 0.00], *p* = 0.037). Results are included in Supporting Information 12: Table S7 and Figure 5.

Mediation analyses were subsequently performed within the sex groups identified by the significant interactions. In the replication dataset, we similarly observed prefrontal mediation: in females, pars triangularis asymmetry—another region within the inferior frontal gyrus—significantly mediated the BMI–treatment response relationship, accounting for 46.4% of the total effect (ACME: *p* = 0.0012). In contrast, supramarginal gyrus asymmetry did not show a significant indirect effect in discovery males. Together, these findings point toward a replicable role of prefrontal cortical asymmetry in linking higher BMI to poorer antidepressant outcomes, with potential sex differences in the precise subregional involvement. Results are included in Supporting Information 12: Table S7 and Supporting Information 13: Table S12, and Figure 4.

### 3.5. Robustness to Additional Covariates

As a stringent test of robustness, we re‐analyzed the primary associations in the replication dataset while adjusting for the comprehensive set of covariates, including dietary patterns (PC1 and PC2), physical activity, sleep duration, educational level, and illness duration. Under this extensive covariate control, the negative association between higher BMI and poorer treatment response remained statistically significant (*b* = −0.986, SE = 0.48, *t* (155) = −2.04, *p* = 0.0435, Supporting Information 9: Table S10), confirming the robustness of the BMI–treatment response association.

Moreover, the mediation effect of cortical asymmetry in the pars triangularis region persisted, showing a significant indirect effect (ACME: *b* = −0.076, 95% CI [−0.180, −0.01], *p*  = 0.031; Supporting Information 14: Table S13 for full statistical results). These findings demonstrate that the mediating role of cortical asymmetry in the relationship between BMI and antidepressant treatment response is robust and not attributable to confounding by the measured lifestyle and socioeconomic factors.

### 3.6. Gene Enrichment Highlights Cytoplasmic Functions

After applying Bonferroni correction, we identified 1149 genes associated with transcriptomic expression in the rostral middle frontal region and 5723 genes in the pars opercularis region, with 432 genes overlapping. When comparing these genes to those reported by Gandal et al. [36] as upregulated or downregulated in MDD, we found 48 overlapping genes. We then performed gene enrichment analysis using DAVID (version 6.8) to explore functional categories associated with these genes. After applying the Bonferroni correction, we observed significant enrichment in the “UP KW CELLULAR COMPONENT” category for the term “KW‐0963~Cytoplasm.” This enrichment included 24 genes (fold enrichment = 1.81, *p*  < 0.05).

## 4. Discussion

Our investigation identifies a neuroanatomical signature linking metabolic health to psychiatric treatment outcomes. We observed that higher BMI is associated with a pronounced leftward shift in prefrontal cortical thickness asymmetry, supporting the “*right brain hypothesis*” in obesity. Critically, this BMI‐driven asymmetry was robustly associated with poorer antidepressant response and acted as a significant mediator in the relationship between BMI and treatment outcome. These findings suggest that structural hemispheric imbalance in the PFC may be a key neurobiological mechanism underlying treatment resistance in patients with comorbid MDD and obesity.

### 4.1. BMI and Prefrontal Asymmetry

The PFC is intricately linked to higher BMI or obesity, both as a contributor to and a consequence of the condition. On one hand, the PFC is critical for dietary control. Neuroimaging studies have shown that individuals affected by overweight and obesity exhibit structural abnormalities, hypoactivity, and disrupted functional connectivity in the PFC, particularly in the dorsolateral PFC (DLPFC) [37, 38]. NIBS techniques targeting the DLPFC have demonstrated efficacy in reducing food cravings and intake [39, 40]. On the other hand, PFC dysfunction may also arise as a consequence of obesity. Prolonged consumption of calorie‐dense foods has been shown to disrupt reward sensitivity and impair PFC functionality, leading to observable deficits in individuals with obesity [41].

Our observation of a leftward shift in asymmetry aligns with previous reports of reduced right frontal thickness in obesity [15, 16]. This structural pattern may reflect a “hypo‐functional” right PFC, a region essential for inhibitory control and dietary restraint. The convergence of these findings underscores the complex role of prefrontal asymmetry: it may serve as both a vulnerability marker for obesity and a scaffold for the cognitive deficits observed in depression. This same prefrontal region is also central to the pathophysiology of depression, as we discuss next.

### 4.2. PFC and Treatment Outcomes

The PFC is one of the regions most consistently impaired in MDD [42]. Structural and functional abnormalities in the PFC have been widely reported in both individuals with current MDD and those at increased vulnerability. The PFC is crucial for emotional regulation and cognitive control. For instance, an fMRI study by Grimm et al. [21] linked asymmetry in DLPFC to negative emotional judgment in severe MDD. Furthermore, TMS targeting the left DLPFC improved emotion regulation compared to sham [43].

The PFC is also among the brain regions most sensitive to the detrimental effects of stress exposure [44]. The right PFC is vital for moderating sensitivity to stress and social pressures and is linked to heightened stress sensitivity and impaired emotional regulation. Young individuals experiencing loss‐of‐control eating often show reduced activation in the DLPFC and ventromedial PFC during acute psychosocial stress. This reduced activation suggests an exacerbation of stress sensitivity and impaired emotional regulation in response to negative peer feedback [45]. In summary, BMI‐induced prefrontal asymmetry may influence how individuals respond to stress, with a pronounced leftward shift potentially leading to greater emotional sensitivity. This sensitivity could predispose individuals to depression by impairing emotional regulation, disrupting neurotransmitter balance, and fostering negative coping mechanisms. These findings suggest that BMI‐related PFC deficits may contribute to MDD risk, particularly in stressful environments.

### 4.3. The Role of Prefrontal Asymmetry in Treatment Outcomes

Our finding that prefrontal asymmetry mediates the link between BMI and treatment response offers a structural explanation for antidepressant resistance. Hemispheric balance in the PFC is crucial for emotional regulation and is a well‐established correlate of therapeutic success. For instance, sMRI studies indicate that successful antidepressant treatment is often accompanied by the normalization of prefrontal asymmetry [46]. However, our data suggest that elevated BMI induces a “structural rigidity” (i.e., a pronounced leftward shift) that may impede this normalization process.

This structural finding converges with functional evidence regarding treatment resistance. Neurostimulation protocols (e.g., rTMS) are based in part on the concept that restoring hemispheric balance may contribute to mood regulation [47, 48]. Moreover, distinct patterns of frontal lateralization have been observed between responsive and treatment‐resistant patients, with resistance often linked to specific disruptions in left‐hemisphere dynamics [49, 50]. Consequently, the BMI‐related asymmetry observed in our cohort may represent a distinct neurobiological phenotype of depression that is inherently less responsive to conventional antidepressant treatments.

### 4.4. Network‐Level Replication and Prefrontal Specificity

A strength of this study is the replication of the mediation effect across two independent cohorts. Although the specific subregions in which significant mediation was observed varied between cohorts (rostral middle frontal/pars opercularis in discovery vs. pars triangularis in replication), they are anatomically adjacent (pars opercularis, Brodmann area 44; pars triangularis, Brodmann area 45; and rostral middle frontal, Brodmann area 46) and functionally integrated within the executive control network [51]. This variance is common in neuroimaging replication studies and likely reflects the continuous nature of cortical topography. The convergence of results within the same functional network strengthens the conclusion that prefrontal asymmetry is the relevant neural substrate.

### 4.5. Sex‐Specific Neuroanatomical Pathways

Beyond this common frontal pattern, our results reveal distinct sex‐specific neuroanatomical pathways linking BMI to poor treatment outcome. In females, the pathway was characterized by BMI‐related asymmetry in the inferior frontal gyrus (pars triangularis), which robustly mediated the link to poorer outcomes. In males, however, asymmetry in the supramarginal gyrus, while associated with both higher BMI and poorer response, did not mediate their relationship. This suggests distinct neurobiological interfaces: in females, elevated BMI may compromise treatment efficacy through circuits supporting verbal–emotional regulation, whereas in males, it presents alongside, but not necessarily via, alterations in regions involved in perceptual‐integrative processing. The absence of mediation in males, despite the significant association, warrants further investigation in larger samples to determine whether this reflects a true lack of mechanistic involvement or insufficient statistical power to detect a smaller effect. This fundamental divergence underscores sex as a critical variable in the neuroscience of depression.

### 4.6. Putative Molecular Mechanisms: The Cytoplasmic Link

Gene enrichment analysis revealed significant enrichment for genes involved in cytoplasmic and metabolic signaling within the implicated regions. These genes regulate metabolic functions related to weight control while also influencing brain signaling pathways involved in mood regulation and depression. Their involvement in inflammatory responses may further link them to increased MDD risk and diminished treatment efficacy.

This suggests that the macroscopic structural asymmetry observed via MRI may be underpinned by cellular metabolic dysfunction. Chronic low‐grade inflammation and metabolic stress associated with high BMI can disrupt cytoplasmic signaling pathways [52], potentially leading to neuroinflammation and reduced neuroplasticity [53]. These molecular alterations may impair the brain’s ability to reorganize in response to antidepressant treatment, linking systemic metabolic health to neural treatment resistance.

### 4.7. Clinical Implications

These findings have translational potential for precision psychiatry. Given that BMI‐related prefrontal asymmetry predicts poor response to standard antidepressants, patients with this profile might benefit from alternative strategies. For example, neuromodulation techniques (e.g., TMS) that specifically target hemispheric rebalancing—such as excitatory stimulation of the right DLPFC or inhibitory stimulation of the left—could be theoretically prioritized for patients with MDD and obesity. In addition, concurrent metabolic interventions (e.g., weight management programs) may complement neuromodulation by addressing the underlying metabolic driver of this neurostructural phenotype.

### 4.8. Methodological Strengths

This study has several strengths. The discovery‐confirmation design enhances the generalizability of the findings, ensuring their applicability across diverse populations. Additionally, the longitudinal cohort, with data collected at baseline and after 12 weeks, strengthens the temporal analysis and provides valuable insights into the progression and treatment response in MDD patients. Furthermore, in the deep‐phenotyped replication cohort, we were able to control for several potential confounding factors, including lifestyle variables (diet, sleep, physical activity), socioeconomic status, and illness duration. While these detailed variables were not available in the discovery dataset, the persistence of our core findings in the replication dataset under this comprehensive confounder adjustment strengthens the validity and robustness of these findings.

### 4.9. Limitations

Several limitations should be considered when interpreting these findings. First, while the total sample size (*n* = 312) provided sufficient power to detect the reported effects, it remains modest for performing detailed stratification analyses (e.g., by medication type or specific genetic risk scores). Larger, multi‐site cohorts would allow for more granular subgroup investigations.

Second, although BMI is the standard clinical metric for weight classification, it is a surrogate measure that does not distinguish between muscle mass and adipose tissue, nor does it quantify visceral adiposity. Future studies should incorporate more direct metabolic phenotypes, such as waist circumference [54], body composition, or cumulative BMI exposure [55], to better isolate the specific metabolic drivers of the observed neural alterations.

Third, the gene enrichment analysis relies on spatial correlations with the AHBArather than patient‐specific transcriptomic data. While this “virtual histology” approach is a widely accepted method for hypothesis generation, it assumes a fixed transcriptional architecture and cannot capture the dynamic gene–environment interactions occurring within our specific MDD cohort.

Fourth, a key limitation concerns causal inference in our mediation model. While our study benefits from temporal precedence (BMI and brain asymmetry were measured at baseline, prior to treatment, and used to predict subsequent response at week 12), the relationship between BMI and brain asymmetry remains cross‐sectional. Therefore, while the direction from baseline brain measures to future outcome is plausible, we cannot definitively establish whether higher BMI causes altered asymmetry, whether pre‐existing asymmetry influences weight regulation, or whether both are influenced by shared genetic or environmental confounders. The mediation analysis should thus be interpreted as revealing a statistically robust and theoretically plausible neuroanatomical pathway linking adiposity to treatment resistance, rather than as proof of a strict causal sequence.

Finally, the observed effect sizes (partial *r* ≈ 0.2–0.3) were small–to–moderate. However, these magnitudes are consistent with complex brain–behavior phenotypes reported in large‐scale consortia such as ENIGMA [18, 56]. Rather than diminishing the significance of the findings, these effect sizes highlight the multifactorial nature of treatment response, where brain structure is one of several contributing biological factors.

## 5. Conclusions

This study identifies a specific neurostructural pathway linking elevated BMI to antidepressant treatment resistance. We demonstrate that high BMI is associated with a pronounced leftward shift in prefrontal cortical asymmetry, which significantly mediates poorer clinical outcomes. Moreover, we identified sex‐specific neuroanatomical circuits linking BMI to poor response, underscoring the importance of biological sex in this pathway. These findings suggest that metabolic dysregulation may compromise the hemispheric balance required for therapeutic recovery. Clinically, our results highlight the potential of prefrontal asymmetry as a stratification biomarker and advocate for precision medicine approaches—such as asymmetry‐targeted neuromodulation or concurrent metabolic interventions—to enhance efficacy in patients with comorbid depression and obesity.

## Funding

This study was supported by STI2030‐Major Projects (2021ZD0200600), the National Key Research & Development Program of China (2016YFC1307200), National Natural Science Foundation of China (82471576), Beijing Research Ward Excellence Program (BRWEP2024W072120106), Beijing High‐Level Innovation and Entrepreneurship Talent Support Program‐Dengfeng Project (G202512066), Research Fund from Beijing Anding Hospital (YG202302), and Special fund of Beijing Municipal Science and Technology Commission (Z171100000117004).

## Conflicts of Interest

The authors declare no conflicts of interest.

## Supporting Information

Additional supporting information can be found online in the Supporting Information section.

## Supporting information


**Supporting Information 1** Table S1. Inclusion and Exclusion Criteria for Discovery Dataset and Replication Dataset.

**Supporting Information 2** Table S2. Imaging Acquisition Parameters for Discovery Dataset and Replication Dataset.

**Supporting Information 3** Figure S1. Principal Component Analysis of Dietary Variables.

**Supporting Information 4** Table S3. BMI‐by‐Sex Interaction Effects on Cortical Asymmetry in the Discovery Dataset.

**Supporting Information 5** Table S8. BMI‐by‐Sex Interaction Effects on Cortical Asymmetry in the Replication Dataset.

**Supporting Information 6** Table S4. Main Effects of BMI on Cortical Asymmetry in the Discovery Dataset.

**Supporting Information 7** Table S9. Main Effects of BMI on Cortical Asymmetry in the Replication Dataset.

**Supporting Information 8** Table S5. Associations Between BMI and Treatment Response in the Discovery Dataset.

**Supporting Information 9** Table S10. Associations Between BMI and Treatment Response in the Replication Dataset.

**Supporting Information 10** Table S6. Associations Between Cortical Thickness Asymmetry and Treatment Response in the Discovery Dataset.

**Supporting Information 11** Table S11. Associations Between Cortical Thickness Asymmetry and Treatment Response in the Replication Dataset.

**Supporting Information 12** Table S7. Mediation Results in the Discovery Dataset.

**Supporting Information 13** Table S12. Mediation Results in the Replication Dataset.

**Supporting Information 14** Table S13. Mediation Results After Controlling for Covariates in the Replication Dataset.

## Data Availability

The data that support the findings of this study are available on request from the corresponding author. The data are not publicly available due to privacy or ethical restrictions.
