# Correction to “Prefrontal Structural Asymmetry Mediates Body Mass Index and Treatment Response in Major Depressive Disorder”

**DOI:** 10.1155/da/9875465

**Published:** 2026-09-15

K. Qiao, Z. Zang, Y. Wang, et al., “Prefrontal Structural Asymmetry Mediates Body Mass Index and Treatment Response in Major Depressive Disorder,” *Depression and Anxiety* 2026 (2026): 9924894, https://doi.org/10.1155/da/9924894.

In the article titled “Prefrontal Structural Asymmetry Mediates Body Mass Index and Treatment Response in Major Depressive Disorder,” there was an error in the author name in the author list and affiliation. The correct author list and affialition as shown below:

Kaini Qiao, Zhenxiang Zang, Yun Wang, Xiongying Chen, Ke Liu, Zhifang Zhang, Rui Liu, Dong Li, Yanting Luo, Le Xiao, Xuequan Zhu, Jingjing Zhou, Zhi Yang, Gang Wang

Beijing Key Laboratory of Intelligent Drug Research and Development for Mental Disorders; National Clinical Research Center for Mental Disorders; National Center for Mental Disorders; Beijing Anding Hospital, Capital Medical University, Beijing, China

We apologize for these errors.

