# Supplementary material for: Prefrontal Structural Asymmetry Mediates Body Mass Index and Treatment Response in Major Depressive Disorder
Source: Depress Anxiety. 2026 May 25;2026:9924894. doi: 10.1155/da/9924894 (PMC13199996; doi:10.1155/da/9924894)
Supplement: Supplementary file 1 — Supporting Information 1 Table S1. Inclusion and Exclusion Criteria for Discovery Dataset and Replication Dataset. [file DA-2026-9924894-s013.docx]

**Table S1. Inclusion and Exclusion Criteria for Discovery Dataset and Replication Dataset.**

| **Criteria** | **Discovery Dataset** | **Replication Dataset** |
| --- | --- | --- |
| **Inclusion Criteria** | - Age 18-65 years; | - Age 18-65 years; |
|  | - DSM-IV MDD diagnosis; | - DSM-5 MDD diagnosis; |
|  | - No systemic antidepressants or <7 days in last 14 days; | - No systemic antidepressants or <7 days in last 14 days; |
|  | - QIDS-SR16 ≥11 and HAMD-17 ≥14; | - HAMD-17 ≥14; |
|  | - At least primary school education; | - At least primary school education; |
|  | - Preparing to use escitalopram | - Planned treatment with a single antidepressant |
| **Exclusion Criteria** | - History of manic or hypomanic episodes; | - Current or past diagnosis of other major psychiatric disorders (DSM-5), including neurodevelopmental, neurocognitive, and psychotic disorders; |
|  | - History of bipolar disorder, schizophrenia, schizoaffective disorder, or other psychotic disorders; | - Depressive episodes secondary to systemic or neurological diseases; |
|  | - History of drug and alcohol dependence or acute intoxication; | - Severe or uncontrolled systemic diseases; |
|  | - Women who are pregnant or lactating; | - Significant physical or laboratory abnormalities; |
|  | - Significant risk of suicidal behavior (HAMD-17 Item 3 score ≥3); | - MECT, TMS, DBS, or VNS within 3 months prior to screening; |
|  | - Current clinically significant disease; | - Inadequate washout for psychotropic medications (less than 7 half-lives); |
|  | - Previously intolerant or lacked response to escitalopram; | - Pregnancy or lactation, or women unable to use effective contraception or planning to become pregnant within 3 months; |
|  | - Any MRI contraindications | - Participation in other interventional clinical trials within 3 months; |
|  |  | - Any MRI contraindications |
