# Supplementary material for: Prefrontal Structural Asymmetry Mediates Body Mass Index and Treatment Response in Major Depressive Disorder
Source: Depress Anxiety. 2026 May 25;2026:9924894. doi: 10.1155/da/9924894 (PMC13199996; doi:10.1155/da/9924894)
Supplement: Supplementary file 2 — Supporting Information 2 Table S2. Imaging Acquisition Parameters for Discovery Dataset and Replication Dataset. [file DA-2026-9924894-s014.docx]

**Table S2. Imaging Acquisition Parameters for Discovery Dataset and Replication Dataset.**

| **Sets** | **Slice Orientation** | **Number of Slices** | **Repetition Time (ms)** | **Echo Time (ms)** | **Slice Thickness (mm)** | **Flip Angle (degrees)** | **Matrix Size** | **FOV** | **Voxel Size (mm3)** | **Number of Samples** |
| --- | --- | --- | --- | --- | --- | --- | --- | --- | --- | --- |
| **Discovery Dataset** | Sagittal | 192 | 2530 | 1.85 | 1 | 9 | 256 x 256 | 256 x 256 | 1 x 1 x 1 | 75 |
|  | Transverse | 192 | 2530 | 2.26 | 1 | 9 | 256 x 256 | 256 x 256 | 1 x 1 x 1 | 32 |
| **Replication Dataset** | Sagittal | 192 | 2530 | 4.21 | 1 | 7 | 256 x 256 | 256 x 256 | 1 x 1 x 1 | 205 |

Note: FOV, Field of View.
