# Supplementary material for: Prefrontal Structural Asymmetry Mediates Body Mass Index and Treatment Response in Major Depressive Disorder
Source: Depress Anxiety. 2026 May 25;2026:9924894. doi: 10.1155/da/9924894 (PMC13199996; doi:10.1155/da/9924894)

Figure S1. Principal Component Analysis of Dietary Variables.

PCA of dietary variables showing (A) scree plot, (B) variable contribution biplot, and (C) individual contribution percentages. Two principal components (PC1: 50.9%; PC2: 25.9%; cumulative: 76.8%) capture 76.8% of dietary variance. PC1 represents a general high-frequency diet pattern (positive loadings: diet_high_fat, 0.573; diet_high_sugar, 0.524; diet_high_salt, 0.613), while PC2 primarily captures probiotic consumption (diet_probiotics, -0.922). PC1 captures a general "unhealthy diet" pattern, while PC2 reflects a specific "probiotic vs. other foods" contrast pattern.

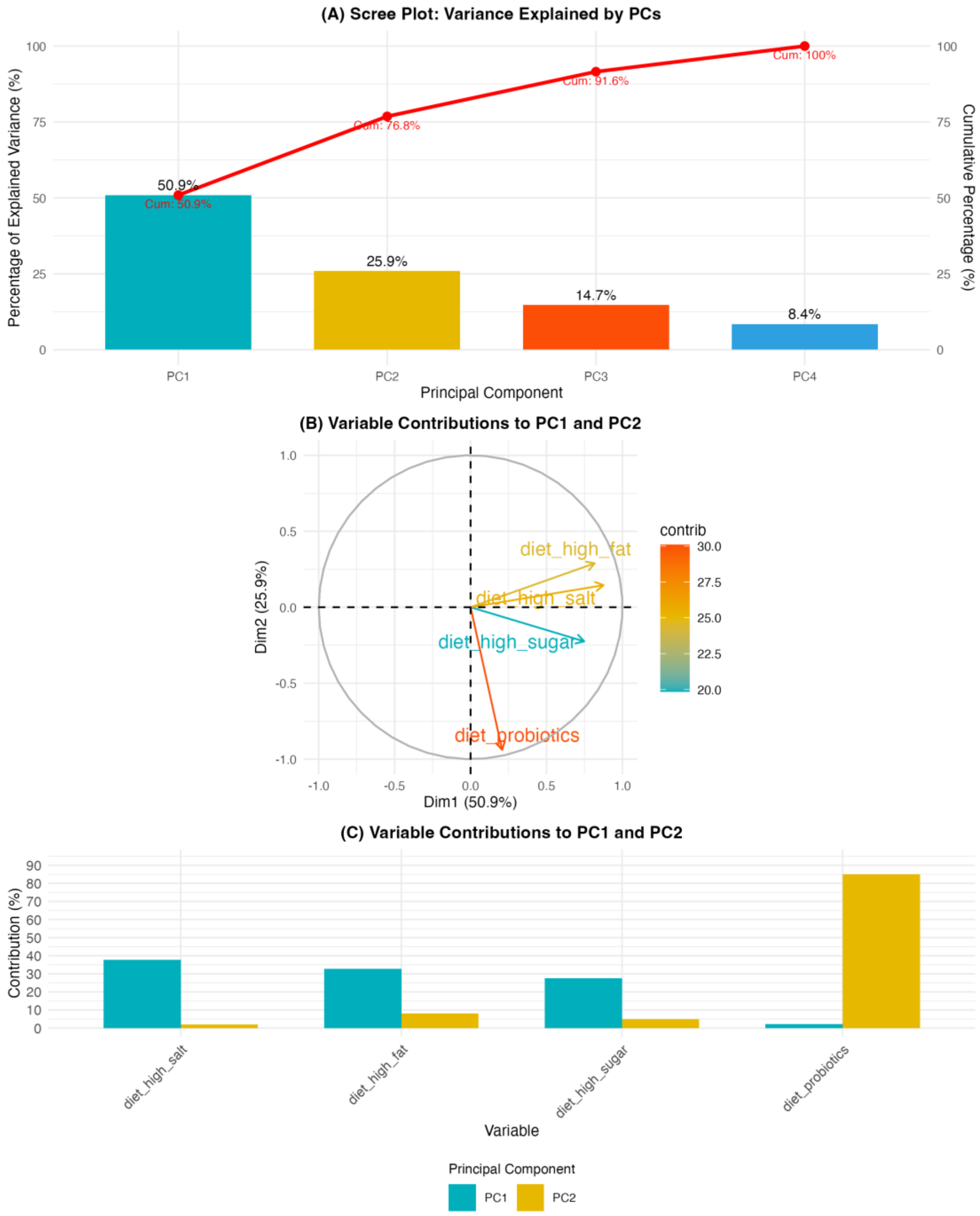

Supplement: Supplementary file 3 — Supporting Information 3 Figure S1. Principal Component Analysis of Dietary Variables. [file DA-2026-9924894-s001.pdf]
