# Supplementary material for: Prefrontal Structural Asymmetry Mediates Body Mass Index and Treatment Response in Major Depressive Disorder
Source: Depress Anxiety. 2026 May 25;2026:9924894. doi: 10.1155/da/9924894 (PMC13199996; doi:10.1155/da/9924894)
Supplement: Supplementary file 4 — Supporting Information 4 Table S3. BMI‐by‐Sex Interaction Effects on Cortical Asymmetry in the Discovery Dataset. [file DA-2026-9924894-s002.docx]

**Table S3. BMI-by-Sex Interaction Effects on Cortical Asymmetry in the Discovery Dataset**

| **Region** | **Interaction_b (n=107)** | **Interaction_p** | **Male_b (n=36)** | **Male_p** | **Female_b (n=71)** | **Female_p** |
| --- | --- | --- | --- | --- | --- | --- |
| **bankssts** | 0.0000 | 0.9789 | -0.0016 | 0.2569 | -0.0016 | 0.1489 |
| **caudalanteriorcingulate** | 0.0007 | 0.7827 | 0.0004 | 0.8494 | 0.0011 | 0.4838 |
| **caudalmiddlefrontal** | 0.0006 | 0.7362 | 0.0004 | 0.7464 | 0.0010 | 0.3283 |
| **cuneus** | 0.0010 | 0.5186 | -0.0009 | 0.4656 | 0.0001 | 0.9176 |
| **entorhinal** | 0.0018 | 0.5230 | -0.0003 | 0.8905 | 0.0015 | 0.3843 |
| **fusiform** | 0.0000 | 0.9993 | -0.0003 | 0.6922 | -0.0003 | 0.6042 |
| **inferiorparietal** | 0.0004 | 0.7253 | -0.0008 | 0.3398 | -0.0004 | 0.4976 |
| **inferiortemporal** | 0.0011 | 0.2802 | -0.0007 | 0.4312 | 0.0005 | 0.4559 |
| **isthmuscingulate** | -0.0025 | 0.1422 | 0.0019 | 0.1619 | -0.0006 | 0.5611 |
| **lateraloccipital** | 0.0008 | 0.4503 | -0.0005 | 0.5491 | 0.0003 | 0.6481 |
| **lateralorbitofrontal** | 0.0003 | 0.8104 | 0.0010 | 0.3971 | 0.0013 | 0.1333 |
| **lingual** | -0.0006 | 0.6397 | 0.0006 | 0.5814 | 0.0000 | 0.9653 |
| **medialorbitofrontal** | 0.0019 | 0.2848 | 0.0005 | 0.7160 | 0.0024 | 0.0272 |
| **middletemporal** | -0.0006 | 0.5871 | -0.0003 | 0.7332 | -0.0009 | 0.1826 |
| **parahippocampal** | -0.0008 | 0.6954 | 0.0020 | 0.2231 | 0.0012 | 0.3371 |
| **paracentral** | -0.0037 | **0.0274*** | 0.0024 | 0.0721 | -0.0013 | 0.1975 |
| **parsopercularis** | -0.0014 | 0.3881 | 0.0025 | 0.0601 | 0.0011 | 0.2861 |
| **parsorbitalis** | -0.0028 | 0.2504 | 0.0047 | 0.0152 | 0.0020 | 0.1799 |
| **parstriangularis** | -0.0017 | 0.3136 | 0.0026 | 0.0625 | 0.0008 | 0.4207 |
| **pericalcarine** | -0.0009 | 0.6652 | 0.0024 | 0.1508 | 0.0015 | 0.2373 |
| **postcentral** | 0.0027 | 0.0741 | -0.0009 | 0.4714 | 0.0018 | 0.0460 |
| **posteriorcingulate** | 0.0013 | 0.3800 | -0.0011 | 0.3461 | 0.0002 | 0.8362 |
| **precentral** | -0.0017 | 0.4613 | 0.0010 | 0.5865 | -0.0007 | 0.6182 |
| **precuneus** | 0.0020 | **0.0320*** | -0.0013 | 0.0751 | 0.0007 | 0.2269 |
| **rostralanteriorcingulate** | 0.0016 | 0.5676 | -0.0010 | 0.6380 | 0.0005 | 0.7467 |
| **rostralmiddlefrontal** | -0.0010 | 0.4204 | 0.0024 | 0.0175 | 0.0014 | 0.0676 |
| **superiorfrontal** | -0.0004 | 0.6831 | 0.0014 | 0.0906 | 0.0010 | 0.1193 |
| **superiorparietal** | 0.0011 | 0.2101 | 0.0001 | 0.9223 | 0.0012 | 0.0302 |
| **superiortemporal** | 0.0006 | 0.5867 | 0.0000 | 0.9941 | 0.0006 | 0.3783 |
| **supramarginal** | 0.0026 | **0.0436*** | -0.0015 | 0.1597 | 0.0012 | 0.1362 |
| **frontalpole** | 0.0030 | 0.2582 | -0.0007 | 0.7568 | 0.0023 | 0.1464 |
| **temporalpole** | 0.0072 | **0.0080**** | -0.0055 | **0.0106*** | 0.0016 | 0.3144 |
| **transversetemporal** | -0.0031 | 0.2246 | 0.0023 | 0.2528 | -0.0008 | 0.6182 |
| **insula** | -0.0017 | 0.0978 | 0.0003 | 0.6920 | -0.0014 | 0.0280 |

*p < 0.05, **p < 0.01, ***p < 0.001.
