# Supplementary material for: Prefrontal Structural Asymmetry Mediates Body Mass Index and Treatment Response in Major Depressive Disorder
Source: Depress Anxiety. 2026 May 25;2026:9924894. doi: 10.1155/da/9924894 (PMC13199996; doi:10.1155/da/9924894)
Supplement: Supplementary file 5 — Supporting Information 5 Table S8. BMI‐by‐Sex Interaction Effects on Cortical Asymmetry in the Replication Dataset. [file DA-2026-9924894-s003.docx]

**Table S8. BMI-by-Sex Interaction Effects on Cortical Asymmetry in the Replication Dataset**

| **Region** | **Interaction_b (n=184)** | **Interaction_p** | **Male_b (n=60)** | **Male_p** | **Female_b (n=124)** | **Female_p** |
| --- | --- | --- | --- | --- | --- | --- |
| **bankssts** | 0.0012 | 0.4280 | -0.0010 | 0.4295 | 0.0002 | 0.8263 |
| **caudalanteriorcingulate** | -0.0015 | 0.4453 | 0.0012 | 0.4788 | -0.0003 | 0.7683 |
| **caudalmiddlefrontal** | 0.0002 | 0.8589 | -0.0008 | 0.3522 | -0.0006 | 0.2524 |
| **cuneus** | 0.0012 | 0.3342 | -0.0014 | 0.1927 | -0.0002 | 0.7837 |
| **entorhinal** | 0.0012 | 0.5511 | -0.0023 | 0.1928 | -0.0010 | 0.3365 |
| **fusiform** | 0.0010 | 0.2365 | -0.0006 | 0.3659 | 0.0003 | 0.4434 |
| **inferiorparietal** | 0.0007 | 0.3598 | -0.0010 | 0.1342 | -0.0003 | 0.4971 |
| **inferiortemporal** | -0.0008 | 0.4072 | 0.0008 | 0.3460 | 0.0000 | 0.9645 |
| **isthmuscingulate** | 0.0003 | 0.7861 | -0.0007 | 0.5146 | -0.0004 | 0.5964 |
| **lateraloccipital** | 0.0005 | 0.5236 | 0.0002 | 0.7607 | 0.0007 | 0.0967 |
| **lateralorbitofrontal** | -0.0012 | 0.2040 | 0.0007 | 0.4004 | -0.0005 | 0.3040 |
| **lingual** | 0.0007 | 0.5070 | -0.0001 | 0.9396 | 0.0006 | 0.2668 |
| **medialorbitofrontal** | 0.0000 | 0.9702 | 0.0009 | 0.4002 | 0.0010 | 0.1614 |
| **middletemporal** | 0.0008 | 0.3295 | -0.0008 | 0.2650 | 0.0000 | 0.9659 |
| **parahippocampal** | -0.0040 | **0.0177*** | 0.0038 | **0.0078**** | -0.0002 | 0.8591 |
| **paracentral** | -0.0019 | 0.1665 | 0.0024 | 0.0391 | 0.0005 | 0.4754 |
| **parsopercularis** | 0.0015 | 0.2243 | -0.0010 | 0.3434 | 0.0005 | 0.4494 |
| **parsorbitalis** | -0.0006 | 0.7093 | 0.0006 | 0.6423 | 0.0000 | 0.9647 |
| **parstriangularis** | 0.0028 | **0.0113*** | -0.0007 | 0.4627 | 0.0021 | **0.0004***** |
| **pericalcarine** | 0.0019 | 0.2652 | -0.0021 | 0.1486 | -0.0002 | 0.8221 |
| **postcentral** | 0.0026 | 0.0637 | -0.0028 | 0.0219 | -0.0002 | 0.8393 |
| **posteriorcingulate** | -0.0003 | 0.8073 | -0.0003 | 0.7898 | -0.0005 | 0.3819 |
| **precentral** | -0.0040 | **0.0414*** | 0.0038 | **0.0235*** | -0.0002 | 0.8494 |
| **precuneus** | 0.0000 | 0.9522 | 0.0003 | 0.6905 | 0.0003 | 0.4580 |
| **rostralanteriorcingulate** | 0.0001 | 0.9525 | 0.0006 | 0.6619 | 0.0007 | 0.4218 |
| **rostralmiddlefrontal** | -0.0002 | 0.8364 | 0.0010 | 0.1506 | 0.0009 | 0.0584 |
| **superiorfrontal** | 0.0000 | 0.9383 | 0.0007 | 0.1579 | 0.0006 | 0.0367 |
| **superiorparietal** | 0.0003 | 0.6683 | -0.0005 | 0.3838 | -0.0002 | 0.5577 |
| **superiortemporal** | 0.0003 | 0.6789 | -0.0012 | 0.1066 | -0.0008 | 0.0733 |
| **supramarginal** | 0.0022 | **0.0176*** | -0.0018 | **0.0219*** | 0.0004 | 0.4347 |
| **frontalpole** | 0.0046 | **0.0320*** | -0.0029 | 0.1066 | 0.0016 | 0.1513 |
| **temporalpole** | 0.0002 | 0.9077 | -0.0003 | 0.8707 | 0.0000 | 0.9659 |
| **transversetemporal** | 0.0004 | 0.8243 | -0.0001 | 0.9563 | 0.0003 | 0.7449 |
| **insula** | -0.0004 | 0.6420 | 0.0005 | 0.4705 | 0.0001 | 0.7783 |

*p < 0.05, **p < 0.01, ***p < 0.001.
