# Supplementary material for: Prefrontal Structural Asymmetry Mediates Body Mass Index and Treatment Response in Major Depressive Disorder
Source: Depress Anxiety. 2026 May 25;2026:9924894. doi: 10.1155/da/9924894 (PMC13199996; doi:10.1155/da/9924894)
Supplement: Supplementary file 6 — Supporting Information 6 Table S4. Main Effects of BMI on Cortical Asymmetry in the Discovery Dataset. [file DA-2026-9924894-s004.docx]

**Table S4. Main Effects of BMI on Cortical Asymmetry in the Discovery Dataset.**

| **Region** | **b** | **SE** | **DF** | **p** | **p(FDR)** | **r** | **95% CI** |  |
| --- | --- | --- | --- | --- | --- | --- | --- | --- |
| **bankssts** | -0.0016 | 0.0009 | 101 | 0.0673 | 0.2972 | -0.181 | -0.3751 | 0.0131 |
| **caudalanteriorcingulate** | 0.0008 | 0.0013 | 101 | 0.5017 | 0.8312 | 0.0669 | -0.1271 | 0.261 |
| **caudalmiddlefrontal** | 0.0008 | 0.0008 | 101 | 0.3294 | 0.6644 | 0.0971 | -0.097 | 0.2911 |
| **cuneus** | -0.0003 | 0.0007 | 101 | 0.7216 | 0.9332 | -0.0355 | -0.2296 | 0.1585 |
| **entorhinal** | 0.0008 | 0.0014 | 101 | 0.5415 | 0.8368 | 0.0609 | -0.1332 | 0.2549 |
| **fusiform** | -0.0003 | 0.0004 | 101 | 0.5134 | 0.8312 | -0.0651 | -0.2592 | 0.1289 |
| **inferiorparietal** | -0.0006 | 0.0005 | 101 | 0.2657 | 0.6022 | -0.1107 | -0.3048 | 0.0834 |
| **inferiortemporal** | 0.0001 | 0.0005 | 101 | 0.9057 | 0.999 | 0.0118 | -0.1823 | 0.2059 |
| **isthmuscingulate** | 0.0003 | 0.0008 | 101 | 0.7029 | 0.9332 | 0.038 | -0.156 | 0.2321 |
| **lateraloccipital** | 0 | 0.0005 | 101 | 0.999 | 0.999 | 0.0001 | -0.1939 | 0.1942 |
| **lateralorbitofrontal** | 0.0012 | 0.0007 | 101 | 0.0879 | 0.2987 | 0.169 | -0.025 | 0.3631 |
| **lingual** | 0.0002 | 0.0006 | 101 | 0.7676 | 0.9332 | 0.0295 | -0.1646 | 0.2235 |
| **medialorbitofrontal** | 0.0017 | 0.0009 | 101 | **0.0487*** | 0.2972 | 0.1947 | 0.0007 | 0.3888 |
| **middletemporal** | -0.0007 | 0.0005 | 101 | 0.2054 | 0.5373 | -0.1258 | -0.3199 | 0.0683 |
| **parahippocampal** | 0.0015 | 0.001 | 101 | 0.1339 | 0.3794 | 0.1487 | -0.0454 | 0.3428 |
| **paracentral** | 0.0001 | 0.0008 | 101 | 0.9491 | 0.999 | 0.0064 | -0.1877 | 0.2004 |
| **parsopercularis** | 0.0016 | 0.0008 | 101 | **0.0484*** | 0.2972 | 0.195 | 0.0009 | 0.389 |
| **parsorbitalis** | 0.003 | 0.0012 | 101 | **0.0125*** | 0.2125 | 0.2453 | 0.0513 | 0.4394 |
| **parstriangularis** | 0.0015 | 0.0008 | 101 | 0.0787 | 0.2972 | 0.1741 | -0.02 | 0.3681 |
| **pericalcarine** | 0.0018 | 0.001 | 101 | 0.0719 | 0.2972 | 0.1781 | -0.016 | 0.3722 |
| **postcentral** | 0.0009 | 0.0007 | 101 | 0.25 | 0.6022 | 0.1144 | -0.0797 | 0.3084 |
| **posteriorcingulate** | -0.0003 | 0.0007 | 101 | 0.6891 | 0.9332 | -0.0399 | -0.234 | 0.1542 |
| **precentral** | -0.0001 | 0.0011 | 101 | 0.9437 | 0.999 | -0.007 | -0.2011 | 0.187 |
| **precuneus** | -0.0001 | 0.0005 | 101 | 0.9084 | 0.999 | -0.0115 | -0.2055 | 0.1826 |
| **rostralanteriorcingulate** | 0 | 0.0013 | 101 | 0.9776 | 0.999 | -0.0028 | -0.1969 | 0.1913 |
| **rostralmiddlefrontal** | 0.0017 | 0.0006 | 101 | **0.0044**** | 0.1502 | 0.2783 | 0.0843 | 0.4724 |
| **superiorfrontal** | 0.0012 | 0.0005 | 101 | **0.0242*** | 0.2738 | 0.2221 | 0.028 | 0.4161 |
| **superiorparietal** | 0.0008 | 0.0004 | 101 | 0.075 | 0.2972 | 0.1762 | -0.0179 | 0.3703 |
| **superiortemporal** | 0.0004 | 0.0006 | 101 | 0.4853 | 0.8312 | 0.0695 | -0.1245 | 0.2636 |
| **supramarginal** | 0.0002 | 0.0006 | 101 | 0.7388 | 0.9332 | 0.0333 | -0.1608 | 0.2273 |
| **frontalpole** | 0.0012 | 0.0013 | 101 | 0.3322 | 0.6644 | 0.0965 | -0.0976 | 0.2906 |
| **temporalpole** | -0.001 | 0.0013 | 101 | 0.4646 | 0.8312 | -0.0729 | -0.2669 | 0.1212 |
| **transversetemporal** | 0.0004 | 0.0012 | 101 | 0.7686 | 0.9332 | 0.0293 | -0.1647 | 0.2234 |
| **insula** | -0.0008 | 0.0005 | 101 | 0.132 | 0.3794 | -0.1494 | -0.3435 | 0.0447 |

*p < 0.05, **p < 0.01, ***p < 0.001.
