# Supplementary material for: Prefrontal Structural Asymmetry Mediates Body Mass Index and Treatment Response in Major Depressive Disorder
Source: Depress Anxiety. 2026 May 25;2026:9924894. doi: 10.1155/da/9924894 (PMC13199996; doi:10.1155/da/9924894)
Supplement: Supplementary file 7 — Supporting Information 7 Table S9. Main Effects of BMI on Cortical Asymmetry in the Replication Dataset. [file DA-2026-9924894-s005.docx]

**Table S9. Main Effects of BMI on Cortical Asymmetry in the Replication Dataset.**

| **Region** | **b** | **SE b** | **DF** | **p** | **p(FDR)** | **r** | **95% CI** |  |
| --- | --- | --- | --- | --- | --- | --- | --- | --- |
| **bankssts** | -0.0002 | 0.0007 | 179 | 0.8203 | 0.9036 | -0.0170 | -0.1653 | 0.1313 |
| **caudalanteriorcingulate** | 0.0001 | 0.0009 | 179 | 0.9036 | 0.9036 | 0.0091 | -0.1393 | 0.1574 |
| **caudalmiddlefrontal** | -0.0007 | 0.0004 | 179 | 0.1467 | 0.5448 | -0.1083 | -0.2558 | 0.0391 |
| **cuneus** | -0.0005 | 0.0006 | 179 | 0.3625 | 0.6799 | -0.0681 | -0.2161 | 0.0799 |
| **entorhinal** | -0.0014 | 0.0009 | 179 | 0.1364 | 0.5448 | -0.1111 | -0.2585 | 0.0363 |
| **fusiform** | 0.0001 | 0.0004 | 179 | 0.8613 | 0.9036 | 0.0131 | -0.1352 | 0.1614 |
| **inferiorparietal** | -0.0005 | 0.0004 | 179 | 0.1763 | 0.5448 | -0.1010 | -0.2485 | 0.0466 |
| **inferiortemporal** | 0.0002 | 0.0004 | 179 | 0.6496 | 0.8740 | 0.0340 | -0.1142 | 0.1822 |
| **isthmuscingulate** | -0.0004 | 0.0006 | 179 | 0.4310 | 0.6978 | -0.0589 | -0.2070 | 0.0892 |
| **lateraloccipital** | 0.0005 | 0.0003 | 179 | 0.1184 | 0.5448 | 0.1165 | -0.0308 | 0.2638 |
| **lateralorbitofrontal** | -0.0002 | 0.0004 | 179 | 0.6684 | 0.8740 | -0.0321 | -0.1803 | 0.1162 |
| **lingual** | 0.0004 | 0.0005 | 179 | 0.3690 | 0.6799 | 0.0672 | -0.0808 | 0.2152 |
| **medialorbitofrontal** | 0.0010 | 0.0006 | 179 | 0.1051 | 0.5448 | 0.1209 | -0.0264 | 0.2681 |
| **middletemporal** | -0.0002 | 0.0004 | 179 | 0.5854 | 0.8379 | -0.0408 | -0.1890 | 0.1074 |
| **paracentral** | 0.0011 | 0.0006 | 179 | 0.0945 | 0.5448 | 0.1247 | -0.0225 | 0.2718 |
| **parsopercularis** | 0.0001 | 0.0006 | 179 | 0.8858 | 0.9036 | 0.0108 | -0.1376 | 0.1591 |
| **parsorbitalis** | 0.0002 | 0.0007 | 179 | 0.7795 | 0.9036 | 0.0210 | -0.1273 | 0.1692 |
| **pericalcarine** | -0.0007 | 0.0008 | 179 | 0.3460 | 0.6799 | -0.0704 | -0.2184 | 0.0775 |
| **postcentral** | -0.0009 | 0.0006 | 179 | 0.1735 | 0.5448 | -0.1016 | -0.2492 | 0.0459 |
| **posteriorcingulate** | -0.0005 | 0.0005 | 179 | 0.3799 | 0.6799 | -0.0656 | -0.2137 | 0.0824 |
| **precuneus** | 0.0003 | 0.0004 | 179 | 0.4037 | 0.6863 | 0.0624 | -0.0856 | 0.2105 |
| **rostralanteriorcingulate** | 0.0007 | 0.0008 | 179 | 0.3644 | 0.6799 | 0.0678 | -0.0802 | 0.2158 |
| **rostralmiddlefrontal** | 0.0009 | 0.0004 | 179 | **0.0192*** | 0.1630 | 0.1740 | 0.0279 | 0.3200 |
| **superiorfrontal** | 0.0007 | 0.0003 | 179 | **0.0126*** | 0.1630 | 0.1851 | 0.0393 | 0.3308 |
| **superiorparietal** | -0.0003 | 0.0003 | 179 | 0.3424 | 0.6799 | -0.0710 | -0.2189 | 0.0770 |
| **superiortemporal** | -0.0009 | 0.0004 | 179 | **0.0189*** | 0.1630 | -0.1743 | -0.3204 | -0.0283 |
| **temporalpole** | -0.0001 | 0.0008 | 179 | 0.9036 | 0.9036 | -0.0091 | -0.1574 | 0.1392 |
| **transversetemporal** | 0.0002 | 0.0009 | 179 | 0.8054 | 0.9036 | 0.0184 | -0.1299 | 0.1667 |
| **insula** | 0.0002 | 0.0004 | 179 | 0.5388 | 0.8327 | 0.0460 | -0.1022 | 0.1941 |

*p < 0.05, **p < 0.01, ***p < 0.001.
