# Supplementary material for: Prefrontal Structural Asymmetry Mediates Body Mass Index and Treatment Response in Major Depressive Disorder
Source: Depress Anxiety. 2026 May 25;2026:9924894. doi: 10.1155/da/9924894 (PMC13199996; doi:10.1155/da/9924894)
Supplement: Supplementary file 8 — Supporting Information 8 Table S5. Associations Between BMI and Treatment Response in the Discovery Dataset. [file DA-2026-9924894-s006.docx]

**Table S5. Associations Between BMI and Treatment Response in the Discovery Dataset.**

| **Model** |  | **b** | **SE** | **df** | **t** | **p** |
| --- | --- | --- | --- | --- | --- | --- |
| **Basic Model (n=65)** |  |  |  |  |  |  |
| BMI × Sex interaction |  | 3.7865 | 1.6563 | 60 | 2.286 | 0.0258* |
| Simple slope | Females (n=44) | -0.0662 | 1.0006 |  | -0.066 | 0.9475 |
|  | Males (n=21) | -3.8527 | 1.3142 |  | -2.932 | 0.0048** |
| **Controlled Model (n=26)** |  |  |  |  |  |  |
| BMI × Sex interaction |  | 1.1008 | 2.4746 | 19 | 0.445 | 0.6615 |
| Reduced Model |  | -2.2433 | 1.0154 | 20 | -2.2092 | 0.0390* |

**Basic Model:** Treatment Response ~ BMI × Sex + Age

**Controlled Model 1:** Treatment Response ~ BMI × Sex + Age + Dosage + HAMD-17 (baseline)
