# Supplementary material for: Prefrontal Structural Asymmetry Mediates Body Mass Index and Treatment Response in Major Depressive Disorder
Source: Depress Anxiety. 2026 May 25;2026:9924894. doi: 10.1155/da/9924894 (PMC13199996; doi:10.1155/da/9924894)
Supplement: Supplementary file 9 — Supporting Information 9 Table S10. Associations Between BMI and Treatment Response in the Replication Dataset. [file DA-2026-9924894-s007.docx]

**Table S10. Associations Between BMI and Treatment Response in the Replication Dataset.**

| **Model** | **b** | **SE** | **df** | **t** | **p** |
| --- | --- | --- | --- | --- | --- |
| **Basic Model (n=177)** |  |  |  |  |  |
| BMI × Sex interaction | -0.9447 | 1.0589 | 172 | -0.8920 | 0.3735 |
| Reduced Model | -1.0120 | 0.4730 | 173 | -2.1397 | 0.0338* |
| **Control for Medication and baseline HAMD-17 scores (n=96)** |  |  |  |  |  |
| BMI × Sex interaction | -0.0314 | 1.3341 | 89 | -0.0240 | 0.9813 |
| Reduced Model | -2.0873 | 0.5355 | 90 | -3.8980 | 0.0002*** |
| **Control for Diet (pc1, pc2), Exercise, Sleep, Education and Illness Duration (n=169)** |  |  |  |  |  |
| BMI × Sex interaction | -0.9666 | 1.0649 | 154 | -0.9077 | 0.3655 |
| Reduced Model | -0.9855 | 0.4842 | 155 | -2.0353 | 0.0435* |

**Basic Model:** Treatment Response ~ BMI × Sex + Age

**Controlled Model 1:** Treatment Response ~ BMI × Sex + Age + Dosage + HAMD-17 (baseline)

**Controlled Model 2:** Treatment Response ~ BMI × Sex + Age + Diet_pc1 + Diet_pc2 + Exercise + Sleep + Education + Illness Duration
