# Supplementary material for: Prefrontal Structural Asymmetry Mediates Body Mass Index and Treatment Response in Major Depressive Disorder
Source: Depress Anxiety. 2026 May 25;2026:9924894. doi: 10.1155/da/9924894 (PMC13199996; doi:10.1155/da/9924894)
Supplement: Supplementary file 10 — Supporting Information 10 Table S6. Associations Between Cortical Thickness Asymmetry and Treatment Response in the Discovery Dataset. [file DA-2026-9924894-s008.docx]

**Table S6. Associations Between Cortical Thickness Asymmetry and Treatment Response in the Discovery Dataset.**

| **Outcome** |  | **b** | **SE** | **df** | **t-value** | **p-value** |
| --- | --- | --- | --- | --- | --- | --- |
| **rostralmiddlefrontal** |  |  |  |  |  |  |
| BMI × Sex interaction |  | -0.4435 | 1.7475 | 59 | -0.254 | 0.8005 |
| Reduced Model |  | -2.0362 | 0.7782 | 60 | -2.6164 | 0.0112* |
| Controlled Model |  | -1.6296 | 0.7362 | 59 | -2.2135 | 0.0307* |
| **parsorbitalis** |  |  |  |  |  |  |
| BMI × Sex interaction |  | 1.3757 | 1.7758 | 59 | 0.775 | 0.4416 |
| Reduced Model |  | -0.3992 | 0.8171 | 60 | -0.4885 | 0.6269 |
| **superiorfrontal** |  |  |  |  |  |  |
| BMI × Sex interaction |  | -0.9014 | 1.6355 | 59 | -0.551 | 0.5836 |
| Reduced Model |  | -0.2906 | 0.8116 | 60 | -0.3581 | 0.7215 |
| **parsopercularis** |  |  |  |  |  |  |
| BMI × Sex interaction |  | 1.8195 | 1.5539 | 59 | 1.171 | 0.2463 |
| Reduced Model |  | -2.183 | 0.7782 | 60 | -2.8052 | 0.0068** |
| Controlled Model |  | -1.9636 | 0.7195 | 59 | -2.7290 | 0.0084** |
| **medialorbitofrontal** |  |  |  |  |  |  |
| BMI × Sex interaction |  | -0.5185 | 2.2840 | 59 | -0.227 | 0.8212 |
| Reduced Model |  | -0.8541 | 0.8086 | 60 | -1.0562 | 0.295 |
| **paracentral** |  |  |  |  |  |  |
| BMI × Sex interaction |  | 1.1933 | 1.6722 | 59 | 0.714 | 0.4783 |
| Reduced Model |  | -1.4596 | 0.8065 | 60 | -1.8099 | 0.0752. |
| **precuneus** |  |  |  |  |  |  |
| BMI × Sex interaction |  | -1.3658 | 1.7321 | 59 | -0.788 | 0.4336 |
| Reduced Model |  | 0.7702 | 0.8288 | 60 | 0.9292 | 0.3564 |
| **supramarginal** |  |  |  |  |  |  |
| BMI × Sex interaction |  | -4.5182 | 1.7672 | 59 | -2.557 | 0.0132* |
| Simple slope | Females (n=44) | -0.3005 | 0.9205 |  | -0.326 | 0.7453 |
|  | Males (n=21) | 4.2178 | 1.4843 |  | 2.842 | 0.0062** |
| Controlled Model |  | -4.7442 | 1.5905 | 58 | -2.983 | 0.0042** |
| Simple slope | Females (n=44) | -0.3419 | 0.8280 |  | -0.413 | 0.6812 |
|  | Males (n=21) | 4.4023 | 1.3359 |  | 3.295 | 0.0017** |
| **temporalpole** |  |  |  |  |  |  |
| BMI × Sex interaction |  | -0.6254 | 1.6411 | 59 | -0.381 | 0.7045 |
| Reduced Model |  | 0.5516 | 0.8095 | 60 | 0.6814 | 0.4982 |

**BMI × Sex interaction model:** Treatment Response ~ Asymmetry × Sex + Age | site;

**Reduced Model:** Treatment Response ~ Asymmetry + Age + Sex | site;

**Controlled Model:** Treatment Response ~ Asymmetry ×/+ Age + Sex + HAMD-17 (baseline) | site.
