# Supplementary material for: Prefrontal Structural Asymmetry Mediates Body Mass Index and Treatment Response in Major Depressive Disorder
Source: Depress Anxiety. 2026 May 25;2026:9924894. doi: 10.1155/da/9924894 (PMC13199996; doi:10.1155/da/9924894)
Supplement: Supplementary file 11 — Supporting Information 11 Table S11. Associations Between Cortical Thickness Asymmetry and Treatment Response in the Replication Dataset. [file DA-2026-9924894-s009.docx]

**Table S11. Associations Between Cortical Thickness Asymmetry and Treatment Response in the Replication Dataset.**

| **Outcome** |  | **b** | **SE** | **df** | **t-value** | **p-value** |
| --- | --- | --- | --- | --- | --- | --- |
| **parstriangularis** | |  |  |  |  |  |
| Basic Model | |  |  |  |  |  |
| BMI × Sex interaction | | -2.3916 | 0.9530 | 172 | -2.510 | 0.0130* |
| Simple slope | Females (n=121) | -2.1515 | 0.5422 |  | -3.968 | 0.0001*** |
|  | Males (n=56) | 0.2401 | 0.7803 |  | 0.3080 | 0.7587 |
| Controlled Model | |  |  |  |  |  |
| BMI × Sex interaction |  | -2.3145 | 0.8619 | 171 | -2.685 | 0.0080** |
| Simple slope | Females (n=121) | -1.7973 | 0.4936 |  | -3.641 | 0.0004*** |
|  | Males (n=56) | 0.5172 | 0.7070 |  | 0.732 | 0.4655 |
| **parahippocampal** | |  |  |  |  |  |
| BMI × Sex interaction | | -0.089 | 0.9639 | 172 | -0.092 | 0.9265 |
| Reduced Model | | -0.4287 | 0.457 | 173 | -0.938 | 0.3495 |
| **precentral** |  |  |  |  |  |  |
| BMI × Sex interaction | | 0.4113 | 0.9337 | 172 | 0.441 | 0.6601 |
| Reduced Model | | -0.6794 | 0.4548 | 173 | -1.494 | 0.1371 |
| **supramarginal** | |  |  |  |  |  |
| BMI × Sex interaction | | -1.1564 | 0.9511 | 172 | -1.216 | 0.2257 |
| Reduced Model | | -0.299 | 0.4639 | 173 | -0.644 | 0.5202 |
| **frontalpole** |  |  |  |  |  |  |
| BMI × Sex interaction | | -1.5389 | 1.0481 | 172 | -1.468 | 0.1439 |
| Reduced Model | | -0.5924 | 0.4574 | 173 | -1.295 | 0.197 |
| **superiortemporal** | |  |  |  |  |  |
| BMI × Sex interaction | | -0.2695 | 0.9829 | 172 | -0.274 | 0.7842 |
| Reduced Model | | -0.7099 | 0.4575 | 173 | -1.552 | 0.1226 |
| **rostralmiddlefrontal** | |  |  |  |  |  |
| BMI × Sex interaction | | -0.7054 | 0.9992 | 172 | -0.706 | 0.4812 |
| Reduced Model | | -0.6246 | 0.4638 | 173 | -1.347 | 0.1799 |
| **superiorfrontal** | |  |  |  |  |  |
| BMI × Sex interaction | | 0.5854 | 0.9698 | 172 | 0.604 | 0.5469 |
| Reduced Model | | -0.4022 | 0.4567 | 173 | -0.881 | 0.3796 |

**BMI × Sex interaction model:** Treatment Response ~ Asymmetry × Sex + Age;

**Reduced Model:** Treatment Response ~ Asymmetry + Age + Sex;

**Controlled Model:** Treatment Response ~ Asymmetry × Age + Sex + HAMD-17 (baseline).
