# Supplementary material for: Prefrontal Structural Asymmetry Mediates Body Mass Index and Treatment Response in Major Depressive Disorder
Source: Depress Anxiety. 2026 May 25;2026:9924894. doi: 10.1155/da/9924894 (PMC13199996; doi:10.1155/da/9924894)
Supplement: Supplementary file 12 — Supporting Information 12 Table S7. Mediation Results in the Discovery Dataset. [file DA-2026-9924894-s010.docx]

**Table S7. Mediation Results in the Discovery Dataset.**

| **Effect Type** | Estimate | 95% CI Lower | 95% CI Upper | p-value |
| --- | --- | --- | --- | --- |
| **In Males (n=21)** |  |  |  |  |
| **supramarginal** |  |  |  |  |
| ACME | -0.1755 | -0.6677 | 0.03 | 0.145 |
| ADE | -0.3804 | -0.6994 | 0.02 | 0.063. |
| Total Effect | -0.556 | -1.025 | -0.18 | 0.006** |
| Prop. Mediated | 0.3157 | -0.0832 | 1.04 | 0.148 |
| **Across Sexes (n=65)** |  |  |  |  |
| **rostralmiddlefrontal** |  |  |  |  |
| ACME | -0.103 | -0.217 | -0.01 | 0.022* |
| ADE | -0.125 | -0.449 | 0.16 | 0.387 |
| Total Effect | -0.228 | -0.525 | 0.03 | 0.078. |
| Prop. Mediated | 0.453 | -1.802 | 4.5 | 0.098. |
| **parsopercularis** |  |  |  |  |
| ACME | -0.093 | -0.232 | 0 | 0.037* |
| ADE | -0.135 | -0.415 | 0.11 | 0.286 |
| Total Effect | -0.228 | -0.52 | 0.02 | 0.072. |
| Prop. Mediated | 0.409 | -0.546 | 2.6 | 0.097. |

ACME = average causal mediation effect (indirect effect); ADE = average direct effect; Total Effect = ACME + ADE; Prop. Mediated = proportion of total effect mediated.
