# Supplementary material for: Prefrontal Structural Asymmetry Mediates Body Mass Index and Treatment Response in Major Depressive Disorder
Source: Depress Anxiety. 2026 May 25;2026:9924894. doi: 10.1155/da/9924894 (PMC13199996; doi:10.1155/da/9924894)
Supplement: Supplementary file 13 — Supporting Information 13 Table S12. Mediation Results in the Replication Dataset. [file DA-2026-9924894-s011.docx]

**Table S12. Mediation Results in the Replication Dataset.**

| **Effect Type** | **Estimate** | **95% CI Lower** | **95% CI Upper** | **p-value** |
| --- | --- | --- | --- | --- |
| **In Females (n=121)** |  |  |  |  |
| **parstriangularis** |  |  |  |  |
| ACME | -0.0913 | -0.1759 | -0.03 | 0.0012** |
| ADE | -0.1054 | -0.274 | 0.05 | 0.1844 |
| Total Effect | -0.1967 | -0.3621 | -0.04 | 0.0108* |
| Prop. Mediated | 0.464 | 0.1289 | 1.79 | 0.0120* |

ACME = average causal mediation effect (indirect effect); ADE = average direct effect; Total Effect = ACME + ADE; Prop. Mediated = proportion of total effect mediated.
