# Supplementary material for: Prefrontal Structural Asymmetry Mediates Body Mass Index and Treatment Response in Major Depressive Disorder
Source: Depress Anxiety. 2026 May 25;2026:9924894. doi: 10.1155/da/9924894 (PMC13199996; doi:10.1155/da/9924894)
Supplement: Supplementary file 14 — Supporting Information 14 Table S13. Mediation Results After Controlling for Covariates in the Replication Dataset. [file DA-2026-9924894-s012.docx]

**Table S13. Mediation Results After Controlling for Covariates in the Replication Dataset.**

| **Effect Type** | **Estimate** | **95% CI Lower** | **95% CI Upper** | **p-value** |
| --- | --- | --- | --- | --- |
| **In Females (n=111)** |  |  |  |  |
| **parstriangularis** |  |  |  |  |
| ACME | -0.0759 | -0.1797 | -0.01 | 0.031* |
| ADE | -0.1187 | -0.3179 | 0.08 | 0.234 |
| Total Effect | -0.1946 | -0.3970 | 0.00 | 0.046* |
| Prop. Mediated | 0.3902 | -0.1665 | 2.55 | 0.076. |

ACME = average causal mediation effect (indirect effect); ADE = average direct effect; Total Effect = ACME + ADE; Prop. Mediated = proportion of total effect mediated.
